# A practical guide for translating in-person simulation curriculum to telesimulation

**DOI:** 10.1186/s41077-022-00210-7

**Published:** 2022-05-12

**Authors:** Ilian Cruz-Panesso, Roger Perron, Valérie Chabot, Frédérique Gauthier, Marie-Michèle Demers, Roxane Trottier, Francis Soulières, Laetitia Juste, Shiva Gharavi, Nathalie MacDonald, Amélie Richard, Audrey Boivin, Benoit Deligne, Karine Bouillon, Pierre Drolet

**Affiliations:** 1 Center for learning of clinical attitudes and skills (CAAHC, Centre d’Apprentissage des Attitudes et Habilités Cliniques), Université de Montréal – Campus de Montréal, Montreal, Canada; 2 Undergraduate Medical Studies, Université de Montréal, Montreal, Canada

**Keywords:** Simulation, Telesimulation, COVID-19, Standardized patients, Teledebriefing

## Abstract

This article provides a road map, along with recommendations, for the adoption and implementation of telesimulation at a large scale. We provide tools for translating an in-presence simulation curriculum into a telesimulation curriculum using a combination off-the-shelf telecommunication platform. We also describe the roles and tasks that emerged within the simulation team when planning and delivering a telesimulation curriculum.

**Supplementary Information:**

The online version contains supplementary material available at 10.1186/s41077-022-00210-7.

## Background

The COVID-19 outbreak disrupted traditional structures of medical education. Learners and medical teachers were called to embrace technology both to provide patient care and medical training [1, 2]. Medical schools were urged to provide online learning and integrate simulation, videoconferencing, and virtual technologies to overcome the training needs of students [1, 3–7]. In this context, telesimulation emerged as a key strategy to provide continuity of medical training [3]. These new ways of interacting and providing care transformed the healthcare system including the teaching of medicine [1, 7–9]. The distance education and the online learning methods have been referred to as the new educational paradigm in medical education [1].

### Telesimulation

Telesimulation is an emerging educational modality in which telecommunication and simulation resources are utilized together to build knowledge and provide skills training and/or assessment to learners at an off-site location [10–12]. It allows learners to have real-time interaction with instructors, standardized patients (SPs) [13, 14], and/or mannequins, usually via videoconferencing tools. Telesimulation represents a unique opportunity to expand simulation training beyond the simulation centers and to overcome economic and geographical barriers, allowing not only local but also intercontinental experiences to happen, which otherwise would require more time, effort, and money or that would simply not be feasible [11, 15, 16]. A recent but growing body of evidence suggests that students exposed to telesimulation have comparable learning outcomes to those exposed to standard live simulations [17, 18].

Telesimulation has been used as a training tool in different areas including neonatology [19, 20], emergency medicine [18, 21–23], otorhinolaryngology [24], intensive health care [25], dermatology [26], pediatrics [27], and for teaching laparoscopic skills [28]. It has also been used successfully in the context of remote certification [29, 30] and interprofessional education [31]. However, there has been no evidence or guidance on how to implement a telesimulation curriculum for programs with large cohorts. The purpose of this article, therefore, is to describe a telesimulation curriculum that supported an ambitious curriculum for large cohorts of up to 250 students attending two simulation centers located on two campuses that belong to the University of Montreal during the COVID-19 pandemic. The information provided in this article reflects our own experience and the available evidence-based practice and literature that emerged during the COVID-19 pandemic. The telesimulation curriculum presented in this paper builds upon a variety of simulation workshops addressing communicational and clinical reasoning competences. This article provides a roadmap, along with recommendations, for telesimulation uptake and implementation for other simulation centers wanting to implement telesimulation at a large scale.

## Methods

### The telesimulation curriculum

Part of the in-person SPs simulation program for medical undergraduate students at the University of Montreal was modified and adapted to meet the logistic and learning requirements for a new telesimulation curriculum. Unlike most of the experiences documented in the literature, which describe the use of mannequin-based telesimulation to train technical skills at a distance, the competencies of the telesimulation program reported in this paper focused on communication skills for which the SP method was adopted [13]. The main objective of the telesimulation curriculum described here was to provide communication and clinical reasoning skills training for first, second, and third-year medical students during the COVID-19 pandemic.

**Fig. 1:**
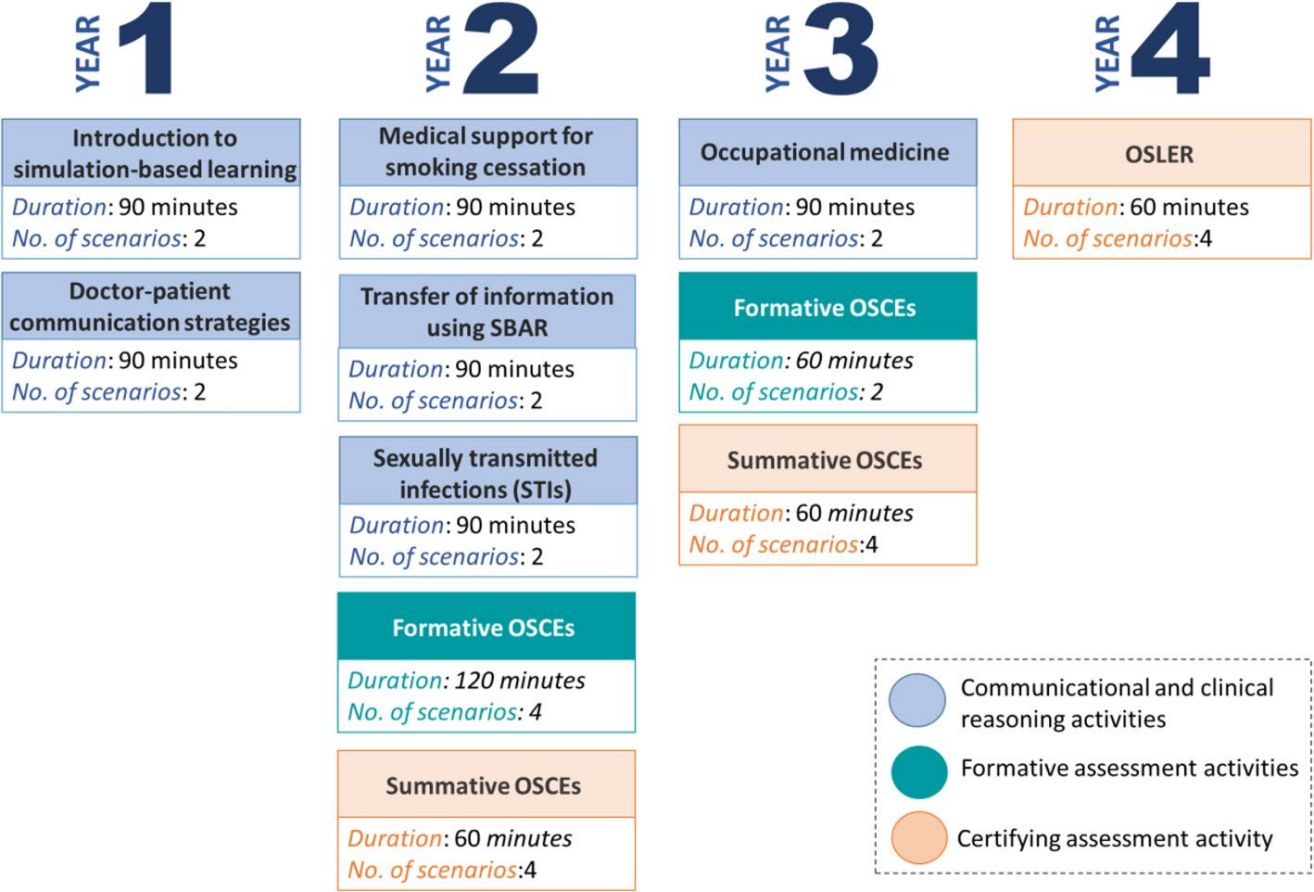
Standardized patient telesimulation curriculum

Telesimulation activities were planned and implemented using a combination off-the-shelf telecommunication platform available at our institution such as Zoom™, Teams™, Moodle™, Qualtrics™, and ExamSoft™. The telesimulation curriculum included six communication training activities as well as two formative assessment activities and three certifying assessment activities (see Fig. 1); however, this paper focuses on describing the training activities. The adaptation of the in-person activities to the telesimulation curriculum consisted of a revision of the learning objectives, the scenarios, and the students’ preparatory activities.

The six communication training activities (see Fig. 1) were chosen out of twelve communication simulations by an academic committee chaired by the assistant dean for undergraduate studies and composed of curriculum officers for each year of the program, the directors of the two simulation centers of the two campuses that belong to the University of Montreal, and simulation and pedagogical experts. Although there were no explicit criteria to select the activities, the following elements played a major role in the decisions made by the committee:

1. Prioritization of activities covering topics that were not taught or discussed in other activities being adapted to an online format such as online problem-based learning (PBL) sessions and online lectures
2. Prioritization of activities that covered topics or taught skills needed for the objective structured clinical examinations (OSCEs)
3. Scenarios requiring a level of fidelity attainable with the telesimulation format used in the present study. For instances, scenarios addressing management of aggressive patients and nakedness were not considered due to the level of realism needed.
4. The number of resources (e.g., instructors, SPs) needed to plan and adapt the activities to the telesimulation format (see Table 1).

**Table 1:** Number of instructors, SPs, students, and iterations per activity

Year	Activity	No. of instructors	No. of SPs	No. of students	No. of iterations
1	• Introduction to simulation-based learning	6	5	298	8
	• Doctor-patient communication strategies	4	4	222	16
2	• Medical support for smoking cessation	4	4	291	16
	• Transfer of information using the SBAR	7	5	297	14
	• Sexually transmitted infections (STIs)	4	5	180	9
3	• Occupational medicine	3	3	306	19

While the curriculum presented in this paper was first proposed as a solution to the training needs of the medical students at the University of Montreal during the pandemic, it is now put forward as an innovative way to partly address the training needs of growing student cohorts expected during the next 3 years in the province of Québec [32].

### Process for planning telesimulation activities

**Fig. 2:**
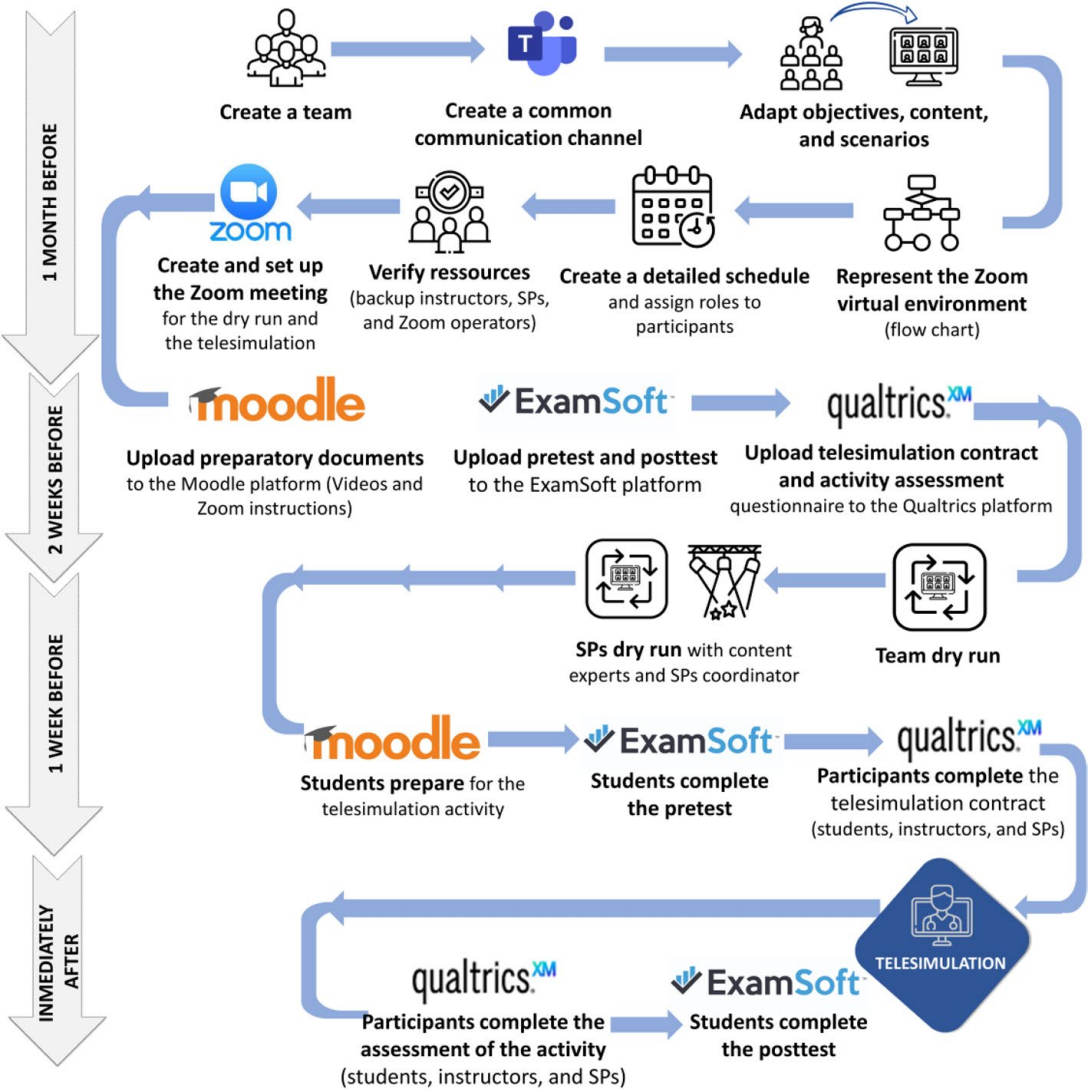
Process for planning and delivering telesimulation activities for big cohorts at the University of Montreal

The process of planning individual telesimulation activities started a month before the activity was scheduled. Figure 2 shows all the different steps and the timeline considered in the planning and the delivery of the telesimulation activities.

#### Assemble a team to plan and run the telesimulation activities

The first step was to assemble a team whose mandate was to plan and run the activities (see Fig. 2). This multidisciplinary team consisted of communication and technology experts, simulation coordinators and administrative managers, medical content experts, simulation instructors, pedagogical advisors, and simulation technicians who coordinated the Zoom™ operations. The number of team members needed for the telesimulation activities varied from nine to fourteen according to the complexity and of the technical needs of each activity (e.g., number of scenarios, number of students, and Zoom™ breakout rooms per session). Further information about the team members’ roles and tasks are described in Additional file 1.

#### Create a common communication channel and prepare for Internet disruptions in advance

A common communication channel was needed to ensure that all team members had the same information at all times but specifically during the planning and the delivery of the telesimulation activity (see Fig. 2). For this purpose, a Teams™ group was set up. This group also served as an alternative communication channel allowing team members to update last-minute changes due to connection issues [33].

#### Revise and adapt objectives, content, and the simulation scenarios

Similar to the general rules applied to the translation of face-to-face courses into online courses [34], the learning outcomes from the in-presence simulation activities to telesimulation did not differ; however, the primary differences and adaptations were made on how the outcomes were achieved. Therefore, the scenarios were reviewed and adapted to the reality of teleconsultations by medical content experts in charge of designing the curriculum for the simulation activities. They were asked to review and adapt the patient’s history and symptoms and add as much detailed information as possible about the pain intensity and the key nonverbal communication behaviors relevant to the case that could be easily conveyed via a video teleconsultation. In some cases, they had been asked that instead of having one long scenario, they develop two short scenarios to allow for rotation of student roles. As the content experts were not necessarily the ones delivering the telesimulations, they were asked to add more detail on the key points that needed to be debriefed. As explained later in this article (see “A test run to test the platform, to clarify roles, to standardize SPs performance, and to prepare for collective debriefings”), content experts also participated in the test-run sessions that were done before each telesimulation, and they supervised the standardization of the SPs.

#### Represent the Zoom™ virtual environment

**Fig. 3:**
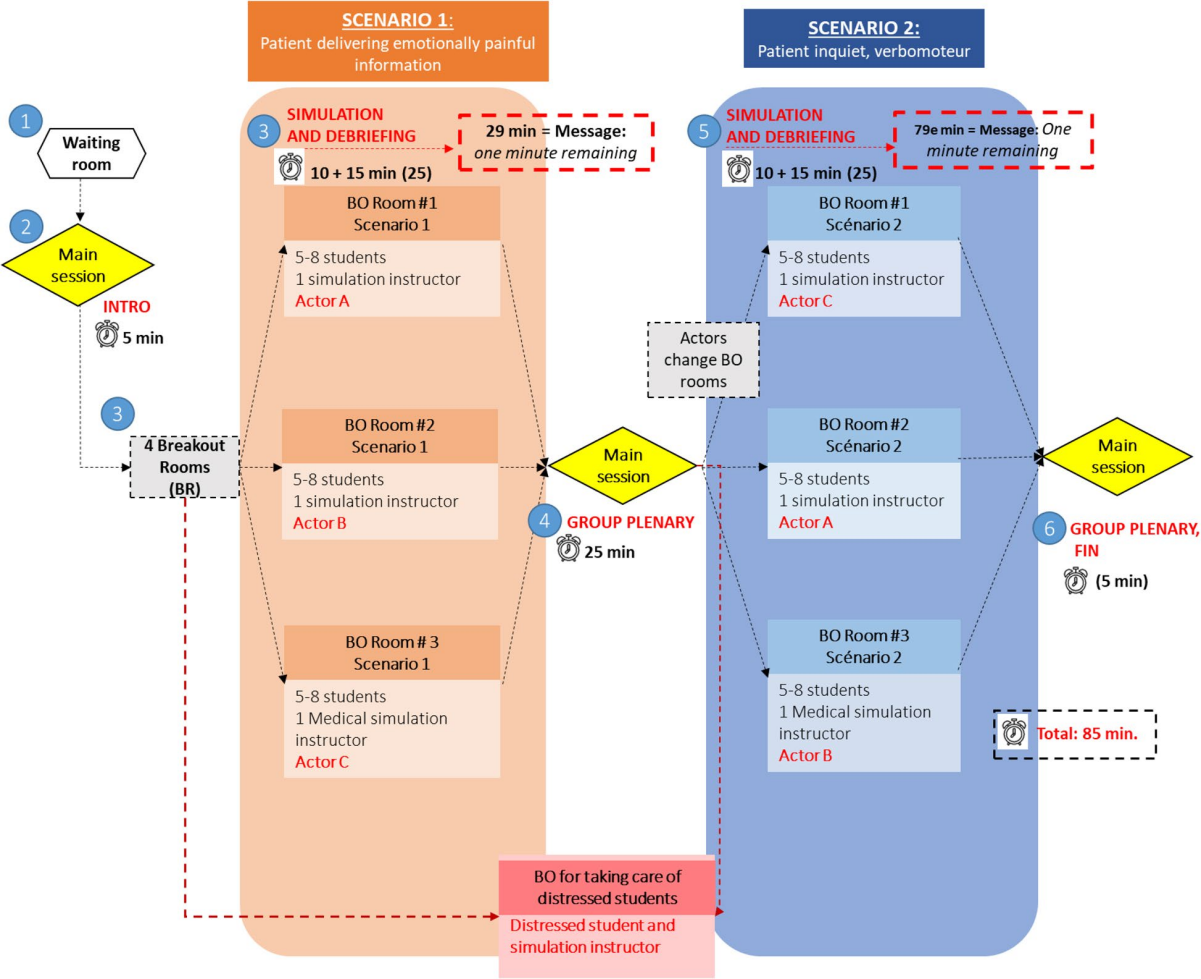
Example of a flowchart of the Zoom™ virtual environment with two scenarios*. ***1** Participants enter the waiting room of the Zoom session 15 min before the simulation starts. Instructors and team members enter main room before the students. **2** Once all the students scheduled for the simulation are in the waiting room, they are all brought to the main session at once. In the main session, there are a 15 min introduction where instructors present the objectives of the activity, the logistics, and the technological instructions. A list with the division of groups and the roles assigned to each participant appears on the screen. **3** The participants are sent to the breakout rooms to encounter the first scenario. The communication scenario should last 10 min, and the teledebriefing 15 min. At 29 min, the Zoom operator sends a message to all the breakout rooms announcing that there is 1 min remaining before the breakout rooms are closed, and all participants are brought to the main session. **4** A plenary with all the instructors initiates and participants are asked to share the key messages discussed in each group. At the end of the plenary, the instructor introduces the context of the second scenario and the assignation of students’ roles. **5** The groups remain the same, and the second scenario starts. The only participants who change breakout room are the SPs. Once again, the scenario should last 10 min, and the teledebriefing last 15 min. Zoom operator sends a cue message announcing that instructors have 1 min before the breakout rooms close. **6** All participants from the different breakout rooms are brought to the main session, and once again, a plenary with all the instructors and students is done. Once the participants are accepted into the main session, the telesimulation should last 85 min in total

The Zoom™ platform was used to create the virtual environment needed for the telesimulation activities. To represent the flow of the Zoom™ virtual environment, each activity was scripted in a flowchart-type diagram (see Fig. 3) that was designed in collaboration between the simulation instructors, the pedagogical advisors, and the Zoom™ operators. These diagrams made it possible to identify the Zoom™ tools necessary for the progression of the activity and the orientation of the students through a path leading them from the waiting room to the debriefing.

Although the flowchart was specific to each activity, they all included four common transitions, namely (a) the virtual waiting room, where the students were first received until all the participants arrived; (b) the main session, where students were briefed about the learning objectives, the different transitions of the activity, the confidentiality policies, the simulation instructions, the division of the groups, and the roles for each participant; (c) then, the flowchart showed the division of groups and the transition to the different scenarios including the timing for each scenario and the timing for debriefing in small groups; and after each scenario, (d) all participants were brought together to a main session where a group debriefing was done sharing the key messages discussed in each group.

A more detailed explanation of the Zoom™ settings needed to conduct telesimulation will be described in “Create and set up the Zoom™ meeting.”

#### Create a detailed schedule and assign roles to participants

**Fig. 4:**
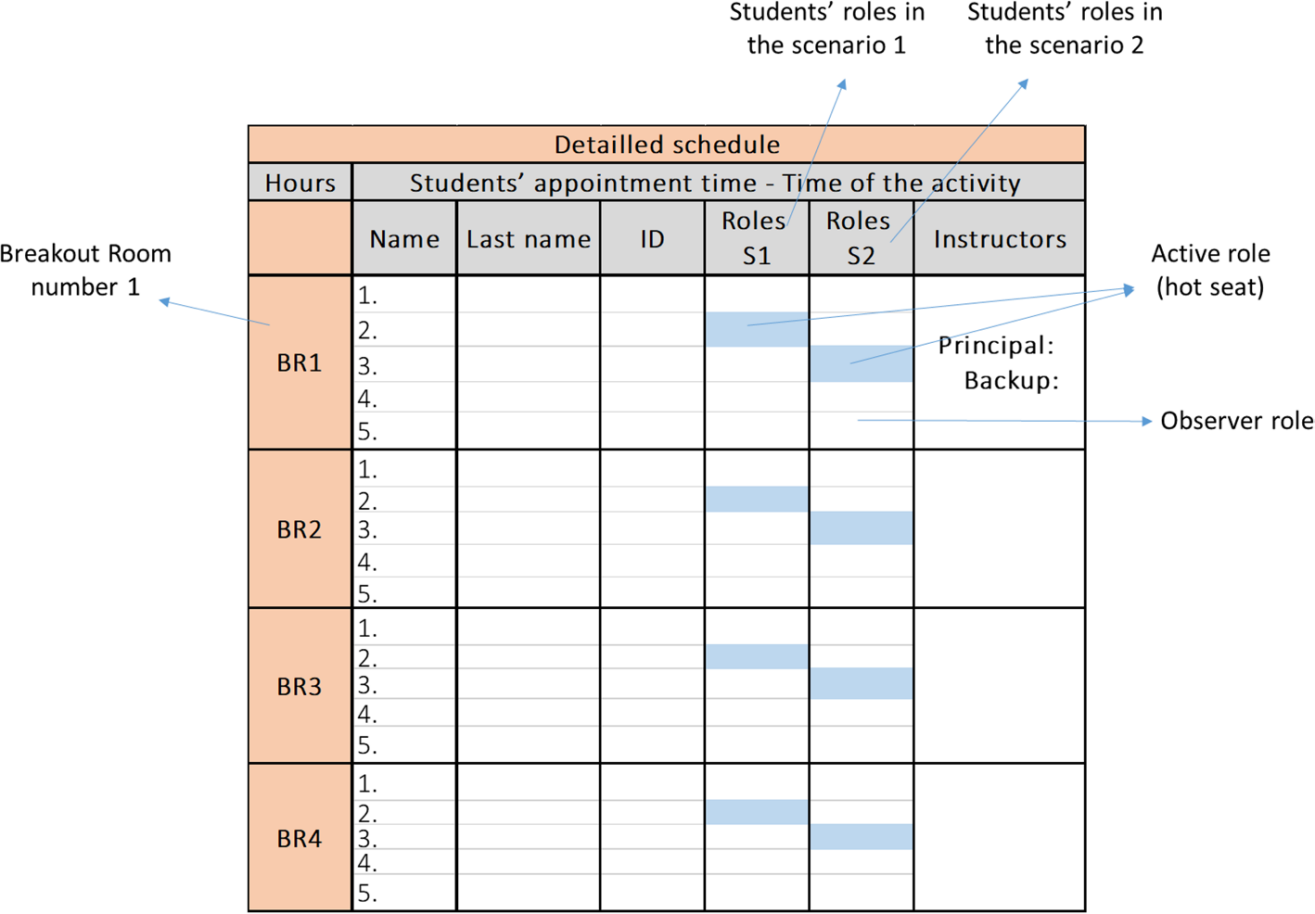
Excel worksheet canvas showing the detailed schedule for the telesimulation activities. This schedule was done 1 month ahead to plan the distribution of the resources. It was also used by the Zoom™ operators during the meeting to manually create the breakout rooms

An Excel™ list of all students from both campuses, instructors, and SPs was organized in alphabetical order. An example of the Excel worksheet is shown in Fig. 4. This list was used to create the students’ appointments specifying the day and times of the activity. Each group of participants, including students, instructors, and SPs, were given group appointments and were asked to sign in 15 min prior to the activity. A group of students between 30 and 40 were cited for each time slot. Telesimulations were run simultaneously for a minimum of four and a maximum of eight groups with a ratio of six to eight students, one SP, and one instructor per group.

Specific roles were assigned to learners beforehand by simulation instructors; however, students were only informed of their role during the introduction to the telesimulation activity. Students’ roles included active or primary physician, resident supervisor, and observer. The telesimulation activities often proposed two scenarios in which students had the opportunity to play different roles. Depending on the scenario, one or two students play an active role, and the others act as observers.

#### Verify resources and prepare for Internet disruptions in advance

**Fig. 5:**
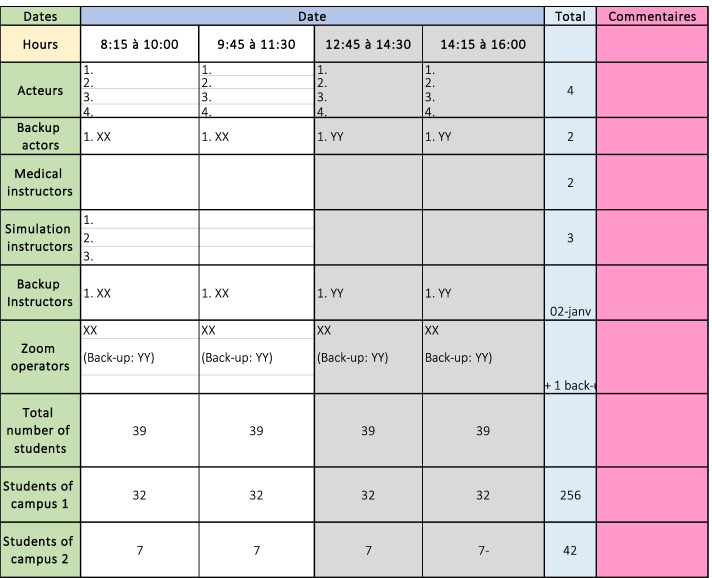
Excel canvas of the list of resources needed for each telesimulation according to the number of students

The quality of telesimulation activities relies enormously on proper anticipation of technical difficulties [12, 35]. To overcome connection or Internet disruption during the telesimulation activities, a backup plan that considered a relay system of simulation instructors, SPs, and Zoom™ operators was designed for each activity. A minimum of two backup instructors, two backup SPs, and 1 backup Zoom™ operator were scheduled for all the telesimulation activities. Backup resources were trained to rapidly intervene in case that one member had connection difficulties. A detailed list of the backup resources needed per day was done in advance and continuously updated as needed (see Fig. 5). The backup system was tested several times before the telesimulations, and it proved useful while running the activities. During the telesimulation activity, when the backup system needed to be deployed, all team members received alerts and messages via the Teams™ channel to prepare accordingly.

Each telesimulation activity was repeated up to nineteen times depending on the number of students and the number of instructors and SPs available (see Table 1). When possible, the instructors and the SPs were the same for all the iterations.

#### Create and set up the Zoom™ meeting

Unfamiliar technological platforms and the difficulties associated with them can deviate participants’ attention, increasing the cognitive workload and affecting their capacity to complete a task [36]. We addressed the issue of unfamiliar technology and the cognitive workload associated with it by having dedicated staff to manage the connection and the Zoom™ operations. Zoom™ operators (a main operator and a backup) were in charge of creating and setting up the learning environment, manage the waiting and the breakout rooms, and help participants to solve technical problems.

A set of pre-meeting features were activated a month before each activity by a Zoom™ operator using an educational account (umontreal.ZoomTM.us). Table 2 describes the functions that were activated for each telesimulation activity.

**Table 2:** Zoom™ pre-meeting settings (1 month before)

Zoom™ settings	Description and rational within the telesimulation curriculum
The waiting room	• It allowed the Zoom™ operator to monitor the number of students present at the moment of the group appointment. Once all the expected students were virtually present in the waiting room and once the instructors and the SPs confirmed that they were ready to start, the operator admitted the students all at once
Breakout rooms	• Groups were virtually created using the breakout room function of the Zoom™ platform. Participants, instructors, and SPs were manually pre-assigned to each breakout room by the Zoom™ operator based on the detailed schedule planned ahead on an Excel sheet (see Fig. 4)
Countdown timer before closing breakout rooms	• The operator sets up this Zoom™ function to give participants a visible countdown of 60 s before bringing everyone to the main session. The countdown also served as hint for the instructors, who needed to wrap up the discussion between students
Allow participants to return to the main session at any time	• This function was disabled, so participants did not have the option to return to the main session by themselves

During the telesimulation activities, all participants, including instructors, SPs, and students, were told not to try to enter or exit the discussion or breakout rooms by themselves, and these features were blocked in advance by the Zoom™ operator. This restriction was intended to allow participants to focus on the activity instead of focusing on the logistics, thus minimizing the cognitive workload and the possibilities of error and/or disconnections. The following instructions were given in the Zoom™ connection guidelines (see more detailed information in “Create and share technical and content preparatory documents”):

*You will never have to take any action to change rooms. Everything, including moving toward a breakout room, will be done by the Zoom*^*TM*^*operators. Do not test the Zoom*^*TM*^*options during the scenario, just do activate or deactivate your microphone as needed while always keeping your camera on.*

##### Accessing the Zoom™ meeting link

All participants, including the students, instructors, SPs, and Zoom™ operators, had access to the link through the Moodle™ institutional platform, which required a login access. Once participants accessed the meeting, the system recognized them and identified them with the username used to log in to the Moodle™ platform. This allowed us to ensure a secure and a disruption-free connection. In addition, it allowed us to identify participants reentering the meeting after being accidentally disconnected and send them to the right breakout room. The following instruction was given in the Zoom™ connection guidelines received by all the participants before the telesimulation (see more detailed information in “Create and share technical and content preparatory documents”):

*If you were inadvertently disconnected from the meeting, reconnect using the link in the Moodle™ platform, and you will be redirected to the room where you were. You do not need to press any buttons to leave the meeting or room*.

Generic usernames and passwords were created for the team members, the simulation instructors, and the SPs with the purpose of easily identifying them in the waiting room and giving them priority access to the meeting. After being identified in the waiting room, team members were transferred by the Zoom™ operators to the main room. Team members were asked to connect 20 min before the students.

#### Create and share technical and content preparatory documents

##### Technical preparatory material

All the participants, including the students, instructors, and actors, received Zoom™ connection tutorials tailored to each group and their specific needs. These guidelines listed the actions that participants needed to do before connecting, including downloading the Zoom™ application to minimize online interferences and accessing the Zoom™ link via the Moodle™ platform. The guidelines also included a checklist of the actions required once the Zoom™ meeting was opened but before the telesimulation started (see Table 3).

**Table 3:** Checklist of the actions required before starting the telesimulation activity

• Disconnect yourself from any virtual private network (VPN)	
• Make sure you are using a stable Internet connection	
• Keep your camera on and your microphone muted and ensure that you have access to the Zoom™ chat function, which will allow you to interact with the instructor and communicate with the Zoom™ operator in case that you encounter a connection problem	
• Set up the participant’s window in the speaker view modality to increase the reality of the meeting with the SP and orient learners to the collaborative environment [37]	
• Do not try to activate any Zoom™ function during the meeting; all the technical operations will be made by the Zoom™ operators	

##### Content preparatory material

To prepare students for the telesimulation activities, we implemented the flipping classroom technique in which students received preparatory material 2 weeks in advance in the form of readings and videos made by the instructors. Videos summarized theory and modelled expert clinicians’ problem-solving and decision-making strategies. They were uploaded to a private YouTube account, and analytics (e.g., most and least watched segments, number and duration of views) was used to monitor how students used the videos to prepare themselves. Although the inclusion of preparatory material was commonly used for our in-person simulations, the use of videos and the analytics were new additions to our pedagogical formula.

#### Prepare and adapt formative assessment strategies

##### Formative assessment before and after the telesimulation

After revising the preparatory material, students were asked to complete a questionnaire intended to assess their level of understanding and integration of the concepts behind the telesimulation. The ExamSoft™ platform was used to disseminate these assessments. An evaluation with questions regarding the notions practised during the simulation was made following the telesimulation.

#### Telesimulation agreement

Before each activity, participants (students, SPs, and instructors) were asked to agree to the terms of a telesimulation agreement, which stipulated that active participation and respectful orientation during the activity were mandatory [38]. The Qualtrics XM™ platform was used to distribute and collect the information from this agreement. The details of the telesimulation agreement are described in the Table 4.

**Table 4:** Information included in the telesimulation agreement

Section	Description
Students’ learning contract	• In this section, students were informed about the format differences between the online and the in-person simulations including the fact that they were going to have pre-assigned roles. They were told about the need to have a professional behavior at all times, to suspend disbelief, and to engage in their role as physicians, which required connecting on time and wearing a white coat as they usually do at the hospital. They were also asked to engage in a respectful relationship with the SPs, as they would in a telemedicine consult
Confidentiality of scenarios	• Participants were informed about their moral obligation not to talk about the scenarios and other participants’ performance. Zoom™ recording functions were disabled for the participants
Security and good Zoom™ practices	• Participants were asked to use a stable connection and to connect via the Moodle™ platform. They were also asked to download the latest Zoom™ version to guarantee that all the functionalities would work when needed
Authorization to record and review video recordings for technical, educational, and research purposes	• Participants were asked to consent (or not) to the use of their data for further technical and/or research purposes

#### A test run to test the platform, to clarify roles, to standardize SPs performance, and to prepare for collective debriefings

A test run was scheduled for each telesimulation 1 or 2 weeks before the activity. It included all participants, except the learners. This practice had different purposes. First, it allowed the team to align the different pieces of information each person held. Second, it served as a rehearsal of the activity with team members playing the role of students. Third, it provided a cross-training practice of the key tasks done by operators and instructors, which allowed a better comprehension of the logistics and facilitated the planning of alternative solutions in case something went wrong. During the test-run sessions, the instructors had the opportunity to interact with the platform as if they were students and revise the flowchart sequence of the telesimulation activity. A discussion about the objectives of the teledebriefing sessions was also included in the test run.

The SPs also participated in a separate test run, which also included the content experts and the SPs coordinator. Its aim was to standardize the nonverbal expressions portrayed in the scenario and some technical aspects such as light, microphone volume, and the video background, which for the most parts was neutral with a white or black wall behind.

#### During the telesimulation activity

All activities began with a presimulation briefing. Instructors first reminded the students of the technical instructions to facilitate communication and technical logistics involved in each activity. Participants were asked to rename themselves (name and last name) avoiding the use of short names. While instructors briefed the students, the Zoom operator manually assigned the students, the instructors, and the SPs to the different breakout rooms. This task was accomplished using the predetermined schedule of the activity as support. To facilitate this task, participants were asked to write in parentheses at the end of their names the role they had in the telesimulation activity including student, actor, actor backup, instructor, instructor backup, Zoom operator, and Zoom operator backup. Although pre-assignation of the breakout rooms is possible through the Zoom™ web portal, this functionality does not allow rotation of the participants into different breakout rooms, which was needed in our case in order to expose students to different scenarios.

During the briefing, students were also presented with an Excel table where they identified their assigned group and their role during the telesimulation. If someone got disconnected from the meeting, the Zoom operator was able to easily reconnect the participant to the right breakout room when looking at the predetermined schedule of the activity. Once the presimulation briefing was done, the simulation instructors informed the Zoom operator, via the Teams™ chat, that they were ready to initiate the breakout rooms. In the breakout rooms, the instructors further clarified what was expected of each role and briefly presented the general information about the patient (e.g., age, context, and reason for consultation).

Before the students initiated the communication with the SP, students were asked to brainstorm about how they expected the scenario to evolve and which questions to ask to address the doctor-patient encounter. The student playing an active role was informed that his/her colleagues were allowed to hint him/her with some questions to facilitate the encounter via the chat box. After each scenario, instructors did a first debriefing in small groups allowing all students to contribute to the discussion from their different roles. Some of the strategies that the simulation instructors used to facilitate discussion and to help learners to overcome perceptions of relational distance are described in Table 5.

**Table 5:** Strategies implemented in SP telesimulation training activities to enhance group reflection during debriefing

Category	Strategy
Open and explicit communication	• All participants were asked to activate the gallery view during the debriefing session • Participants were asked to rename themselves as soon as they enter the main room. This allowed to address them by their names, making more personal the communication • As each participant was assigned to a role, they were asked to share their experience and/or their observations • Input was encouraged by directing questions at certain learners who did not participate spontaneously [37] • To avoid losing nonverbal cues during the teledebriefing, participants were asked to keep their cameras and microphones open to facilitate interaction [39, 40]
Emotional expression	• A protocol to address unpredicted emotional reactions was established. If needed, individuals were invited to join an instructor in a separate breakout room where more detailed follow-up was done
Group cohesion	• Small groups of 6–8 students in which students were asked to participate spontaneously • Students were asked to activate their microphones to participate on a voluntary basis

Following the teledebriefing in small groups, the instructors convened for a common plenary session in which the key messages from each breakout room were shared. One student from each breakout room was chosen to convey the key messages.

As connection disruption could alter the timing of the telesimulation activities, the Zoom™ operators also played the role of timekeepers during the activity. In this role, they were responsible for adjusting the schedule of the telesimulation activity in the event of technical or other difficulties delaying operations. When this happened, Zoom™ operators announced the delays and the strategies to overcome them to all team members through the Teams channel.

#### After the telesimulation

Following each telesimulation activity, team members involved in the telesimulation planning immediately conducted a debriefing session to address the technical and pedagogical aspects that needed improvement. Students, simulation instructors, and SPs were also asked to complete an anonymous survey about the perceived usefulness of the telesimulation activity. The survey consisted of 5-point Likert scale statements (1 = completely disagree, 5 = completely agree) (see Additional file 2). The survey was divided into four categories including preparation and organization of the telesimulation (3 questions), familiarity with the technology used during the telesimulation (10 questions), perceived differences between telesimulation and in-person activities (10 questions), and a global assessment of the experience (5 questions). Data from the usability survey is being currently analyzed.

### Limitations

The telesimulation curriculum presented in this article has been successfully applied to learners (*n* = 2989) with minimal disruption and/or technical difficulties; however, it is important to highlight the fact that telesimulation in the format presented in this paper is time-consuming and more expensive than conducting in-person simulations. Other studies and reports on telesimulation have underlined its feasibility and proposed it as a low-cost alternative to provide training during the pandemic [13]; however, our experience suggests that telesimulation could become quite expensive with large student cohorts (more than 250 learners). The requirements to ensure active and engaged participation of the learners during telesimulation, such as small 6–8 student groups (compared to our typical 15–20 student groups during our in-person simulation), as well as the need for personal backup able to react quickly in case of technical or connection difficulties, all contribute to the necessity of securing appropriate resources. In addition, telesimulation activities, in comparison with in-person simulations, require additional standardization processes (test runs, standardization of SPs) that increase the preparation time needed for simulations.

### Lessons learned and recommendations

The process of deploying telesimulation for big cohorts with minimal disruptions at the University of Montreal required a well crafted and a structured plan that was revised and adapted several times based on strengths and weaknesses. Table 6 shows the most recurrent problems and its possible solutions.

**Table 6:** Technical and logistic problems and its possible solutions

Problem	Solution
Students could not connect at the time they were scheduled	• Students were contacted individually by the secretary of the program, who assigned them a different time and group. In cases where several students experienced the same connection difficulties, they were all scheduled to be the last group of the day
Students enter the Zoom™ meeting and got disconnected or had Internet problems	• Students were instructed to reconnect to the Zoom link via the Moodle platform
Students connecting twice simultaneously using the Internet browser version and the Zoom™ application	• The Zoom operator ejected the students (the 2 duplicates) and told them to wait for the Zoom application to start without clicking the browser version link
SPs and instructors could not connect at the time they were scheduled	• The operator moved the backup SP and/or instructor in the room, and the nonavailable one became the backup when he got power back and join the Zoom meeting
SPs and instructors enter the Zoom™ meeting and got disconnected or had Internet problems	• The operator moved the backup actor and/or instructor in the room
The main Zoom™ operator could not connect and open the meeting	•A generic account for the simulation center was created. This account was used by the Zoom operator to create the links for the telesimulation activities. Other team members with access to the generic were given the co-host role, which allows them to open the meeting in case that the main Zoom operator had connection issues
The main Zoom operator got disconnected or had Internet problems	• For each telesimulation activity, two operators were assigned. One acted as the main operator and the other as backup

Based on the expertise and experience acquired by our team in the design and implementation of the telesimulation curriculum for large student cohorts, we propose some recommendations for teams wanting to reproduce our model (see Table 7).

**Table 7:** Recommendations for other simulation centers wanting to implement telesimulation at a large scale

Category	Recommendations
From a technical point of view — Zoom operators	• Zoom operators need to be familiarized with simulation-based training • Try to keep the plan as simple as possible • Adapt to the ongoing changes (e.g., instructors need more time to finish the session) and adjust the timing in a faster manner • Keep good communication with the team at all times. For instance, communicate with the team via the Teams channel when the time for the different sections has been adjusted based on technical or other difficulties
From a simulation instructor point of view	• Have a good knowledge of the activity (content and logistics) • Standardize briefing and debriefing points among instructors • Keep your role as facilitator and avoid solving technical problems •Promote learner’s participation (attribution of roles ahead of time; call students by their names) • In case of technical problems, communicate with the Zoom operators using the chat in the Teams group. This allows all team members to know the difficulties • Know the alternative plan in case of connection difficulties and/or absences and adapt rapidly • Be mindful of time and follow the protocol described in the flowchart
From a pedagogical point of view	• Revise and adapt the learning objectives to the virtual format. • Keep all learners active at all times. Assign specific aspects to observe to the observer students
From an SPs point of view	• Choose SPs with expertise in simulation and who feel comfortable using technology • Standardize the performance of the SPs (define the corporal and the intensity of the emotional reactions that need to be privileged and emphasized/minimized in a virtual environment)

## Conclusions

Telesimulation has great potential to provide distance training for medical students not only during the COVID-19 pandemic but also as a regular offer; however, adapting in-person simulation activities to a telesimulation format for big cohorts requires a lot of effort and expertise from a multidisciplinary team. Smooth adaptation of the curriculum depends on the coordination of resources and anticipation of difficulties. While a backup system, inluding additional instructors, SPs, and Zoom operators increases the cost and number of human resources required to perform telesimulations, it helps deliver the program on time without the difficulties of reprogramming activities due to connection issues, which could sometimes be more expensive in the case of big cohorts. Further evaluation of the telesimulation curriculum presented in this paper will contribute to determine the full potential of transferring in-presence simulation activities aiming at training communication and clinical reasoning competences to telesimulation.

Although some studies comparing telesimulation versus standard simulation have found no significant differences among these two modalities [17], we recommend that future studies look at performance [17, 18], doctor-patient relationship, and debriefing [40] differences of learners exposed to telesimulation and in-presence simulation. Research is still needed to provide insights about the indirect impacts of using telesimulation. For instance, we need measures about learners’ engagement and the positive and negative effects of cognitive load when using multiple communication channels during telesimulation.

From a pedagogical point of view, telesimulation offers alternative opportunities to engage students, playing the active and the observant role, at all times during the simulation and not only during the debriefing. Telesimulation brings the opportunity to expand different ways of communicating and unveiling the reasoning of participants. For instance, the use of the chat and the input of the observers to the students playing an active role could potentially promote the development of common shared mental models among participants allowing instructors to unveil the reasoning of a group of students even before the debriefing. Having traces of the reasoning of a group of students during the simulation could potentially facilitate self-awareness and thus the reflexive thought process. In our experience, we informally observed that for the most part, students felt comfortable communicating through a chat during the simulation, and that they were more likely to participate in virtual debriefings than in-presence debriefings. Virtual debriefings seem to counteract the dominance of a few vocal students offering a more democratic environment for shy participants. Although the virtual debriefings kept the same length as in-presence debriefings, students often reported, verbally, that virtual debriefings were too short, and that they wanted to continue. Although we foresee that telesimulation offers an opportunity to reshape much of the traditional ways of learning in simulation, we also noted that telesimulations touching on more sensitive communication topics (e.g., harassment) could benefit from an in-presence format where a psychological safety environment and a closer follow-up can be assured. For instance, students who identified with the scenarios or who find difficult to interact with the SPs might purposely disconnect from the activity making it difficult to offer an opportunity to debrief and to address the issues. The affordances of virtual debriefings and the strategies to promote students’ psychological safety in telesimulation context need still to be considered and formally studied.

## Supplementary Information

**Additional file 1.** Roles and specific tasks of the organizing team of the telesimulation curriculum.

**Additional file 2.** Telesimulation usability survey.

## Data Availability

All data generated or analyzed during this study are included in this published article.

## Abbreviations

SPs: Standardized patients
PBL: Problem-based learning
VPN: Virtual private network
