# Supplementary material for: A practical guide for translating in-person simulation curriculum to telesimulation
Source: Adv Simul (Lond). 2022 May 12;7:14. doi: 10.1186/s41077-022-00210-7 (PMC9096760; doi:10.1186/s41077-022-00210-7)
Supplement: Supplementary file 1 — Additional file 1. Roles and specific tasks of the organizing team of the telesimulation curriculum. [file 41077_2022_210_MOESM1_ESM.docx]

**ADDITIONAL FILE 1** - Roles and specific tasks of the organizing team of the telesimulation curriculum

| *Roles and specific tasks of the organizing team of the telesimulation curriculum* | |
| --- | --- |
| **Roles** | **Specific tasks** |
| ***Communication and technology experts*** | - Identify and test the communication platforms suitable to run telesimulation activities with |
| ***Coordinator of the telesimulation activity*** | - Plans and coordinates the tasks of the different members involved in the online activities - Determines the schedule of the telesimulations. - Understands the logistic and the pedagogical structure of the activity in order to ensure that the technical (e.g., platforms) and human resources needed for it are covered - Manages and coordinates communication among the members of the team (e.g., creates online documents that can be edited by all members, creates a Teams^TM^ group) - Divides the group of students according to the available resources - Prepares and coordinates a dry-run with all the participants, except the students, two weeks before the telesimulation activity - Establishes and coordinates a plan B in case that there are technical problems |
| ***Administrative coordinator*** | - Contacts and hires the simulation instructors needed for the telesimulation activities |
| ***Medical content experts*** | Medical content experts   - Revise the pedagogical objectives and adapt them to an online format - Revise and update the content of the preparatory material as needed - Revise, transform and/or write up clinical simulation scenarios adapted to the needs of telesimulation - Revise and update the pretest and posttest questions - Participate in the preparation of SPs |
| ***Simulator instructors*** | Simulation instructors   - Participate in the discussions about the adaptation/transformation of the live simulation activities to telesimulation - Creates the flowchart of the telesimulation and update it as needed - Edit and upload the preparatory material into the institutional Moodle platform - Assign the roles of the students for each scenario before hand and in case there are absent students re-assign roles on the fly as needed - Standardize the teaching elements of the telesimulation activities as needed - Animate the telesimulation activities |
| ***Coordinator of simulated patients’ SPs*** | - Recruits the SPs needed for the online simulations - Trains the SPs in the use of the platforms and tools used to deliver telesimulation activities - Standardizes SPs performance - Collaborates with the medical instructors in the revision of the scenarios |
| ***Pedagogical advisors*** | - Supports the team in the pedagogical revision of the activity in particular the revision of the learning objectives adapted to an online-based learning - Ensure that best simulation-based practices are implemented - Prepare the questionnaires needed to assess the activity from the students’ instructors’, and logistics’ point of view |
| ***Zoom operators*** | - Create and configure the Zoom links with based on the needs of each telesimulation activity - Contributes to the revision and update of the activity flowchart - Create and manages breakout rooms during the telesimulation activity - Manages the time and all the Zoom related operations (e.g., waiting room admissions and communications) during the telesimulation activities. |
