# Supplementary material for: A practical guide for translating in-person simulation curriculum to telesimulation
Source: Adv Simul (Lond). 2022 May 12;7:14. doi: 10.1186/s41077-022-00210-7 (PMC9096760; doi:10.1186/s41077-022-00210-7)
Supplement: Supplementary file 2 — Additional file 2. Telesimulation usability survey. [file 41077_2022_210_MOESM2_ESM.docx]

**ADDITIONAL FILE 2 –** Telesimulation usability survey

Thank you for participated in the CAAHC online simulation training at the University of Montreal. We kindly ask you to complete the following anonymized survey so that we can improve / modify the training.

We anticipate that completing the questionnaire will only take you 5 minutes.

We will ask you to read a series of statements, please express your level of agreement using the following 5-point likert scale:

| **Strongly disagree** | **Disagree** | **Undecided** | **Agree** | **Strongly agree** |
| --- | --- | --- | --- | --- |
| 1 | 2 | 3 | 4 | 5 |

| **Assessment of the telesimulation activity** | | | | | |
| --- | --- | --- | --- | --- | --- |
| **Preparation and organization** | **1** | **2** | **3** | **4** | **5** |
| The time I invested to prepare this telesimulation activity was adequate. |  |  |  |  |  |
| The preparatory information I received (Zoom instructions, experts’ instructional video) prepare me well for the telesimulation activity. |  |  |  |  |  |
| The schedule and flow of the telesimulation activity was clear. |  |  |  |  |  |
| **Familiarity with the technology** |  |  |  |  |  |
| I am familiarized with videoconference application. |  |  |  |  |  |
| I am familiarized with the videoconference application Zoom^TM^ |  |  |  |  |  |
| The Zoom connection was easy and reliable |  |  |  |  |  |
| The online survey regarding the learning contract was easy to access. |  |  |  |  |  |
| Time management while interacting with the Zoom^TM^ platform was efficient. |  |  |  |  |  |
| The sound quality during the telesimulation activity was good. |  |  |  |  |  |
| The video quality during the telesimulation activity was good. |  |  |  |  |  |
| The technical support before and during the activity was good. |  |  |  |  |  |
| I did not have any technical problem during the telesimulation activity. |  |  |  |  |  |
| The technical problems I did encountered during the telesimulation activity did not affect the quality of the activity. |  |  |  |  |  |
| If you did encounter technical problems, please comment and give examples | | | | | |
| **In-presence activities vs. telesimulation activities** |  |  |  |  |  |
| I did spend more time preparing for this telesimulation activity than for a regular in-person simulation activity. |  |  |  |  |  |
| The communication with the standardized patient was not as good in this telesimulation activity as in a regular in-person simulation activity. |  |  |  |  |  |
| The instructor’s feedback was not as good in this telesimulation activity as in a regular in-presence activity. |  |  |  |  |  |
| The evaluation of the patient was more difficult in this telesimulation activity in comparison with in-person simulation activities. |  |  |  |  |  |
| In comparison with an in-presence activity, this telesimualtion activity was short and I needed more time to develop rapport with the patient. |  |  |  |  |  |
| I did not learn as much in this telesimulation activity as I do in in-person simulation activities |  |  |  |  |  |
| The fidelity of the scenario was not as good in this telesimulation activity as in a regular in-person simulation activity. |  |  |  |  |  |
| The telesimulation activity was distracting |  |  |  |  |  |
| I was stressed in this telesimulation activity in particular when I was asked to intervene. |  |  |  |  |  |
| I do not value as much the telesimulation activities as I value in-person simulation activities. |  |  |  |  |  |
| **Global experience** |  |  |  |  |  |
| I am generally satisfied with the telesimulation activity |  |  |  |  |  |
| I would like to participate more in telesimulation activities |  |  |  |  |  |
| I believe that the telesimulation activities should be implemented even after the pandemic |  |  |  |  |  |
| I believe that the telemedicine will become even more relevant in the next years |  |  |  |  |  |
| I would like to have more opportunities to be formed in telesimulation |  |  |  |  |  |
| Could you please mention three or more positive elements of the telesimulation activities? | | | | | |
| Could you please mention three o more aspects that need to be improved during the telesimulation activities? | | | | | |
